# Subacute Cutaneous Lupus Erythematosus Secondary to Intravenous Immunoglobulin Infusions

**DOI:** 10.7759/cureus.63217

**Published:** 2024-06-26

**Authors:** Taylor Fleshman, Ian Depew, William Snider, Shane Cook

**Affiliations:** 1 Dermatology, Marshall University Joan C. Edwards School of Medicine, Huntington, USA

**Keywords:** intravenous immunoglobulin (ivig), chronic inflammatory demyelinating polyneuropathy (cidp), scle, drug-induced scle, cutaneous lupus erythematous, subacute cutaneous lupus erythematosus

## Abstract

Subacute cutaneous lupus erythematosus (SCLE) is a variant of cutaneous lupus erythematosus (CLE) characterized by distinct skin lesions. Clinical manifestations typically include annular or psoriasiform skin lesions, often localized in sun-exposed areas such as the chest and back. The pathogenesis of SCLE is largely unknown, and contributing factors include genetics, environmental exposures, and immunological dysregulation. SCLE may be idiopathic or drug-induced, with common triggers being calcium channel blockers, thiazide diuretics, and terbinafine. Intravenous immunoglobulin (IVIG) treatment, frequently used in various autoimmune conditions, has a rare association with SCLE. We report a case in which this condition arose during IVIG treatment for chronic inflammatory demyelinating polyneuropathy (CIDP). Knowledge of this rare effect is beneficial to all providers who prescribe IVIG, including neurology, rheumatology, and dermatology.

## Introduction

Subacute cutaneous lupus erythematosus (SCLE) is a subtype of cutaneous lupus erythematosus (CLE) which may co-occur with systemic lupus erythematosus (SLE) or as an independent entity [1]. Cutaneous findings in SCLE are most commonly papulosquamous plaques or annular, erythematous plaques with trailing scale in symmetrical, photo-exposed areas [2]. Areas above the neck are usually not involved, which is an important diagnostic clue [1]. Unlike SLE, SCLE is characterized by a lack of systemic manifestations, increased cutaneous findings, and increased photosensitivity [3]. While not fully understood, there seems to be some association between SCLE and SLE [4].

The incidence of CLE is around 4 per 100,000, with SCLE representing approximately 14-15% of those cases [5]. The pathogenesis of SCLE is complex and involves genetic, immunologic, and environmental factors, but most cases are positive for serum anti-Ro/SSA antibodies [6]. Pathology generally reveals interface dermatitis with follicular plugging, hyperkeratosis, lymphocytic infiltrate, mucin deposition in the dermis, and IgG deposition at the dermo-epidermal junction [7]. The development may be idiopathic or drug-induced, with the most common causes being calcium channel blockers, thiazide diuretics, and terbinafine [8-9]. Presented here is a case of SCLE which arose in a patient receiving intravenous immunoglobulin (IVIG) therapy for chronic inflammatory demyelinating polyneuropathy (CIDP). Previous literature includes case reports on IVIG-induced CLE and a six-case series on IVIG-induced SCLE in the setting of CIDP [10-11]. The current case was previously presented as a poster at the 2023 West Virginia Dermatological Society Meeting on August 11, 2023.

## Case presentation

A male in his 50s presented with a nine-month history of a papulosquamous rash on the chest, shoulders, and upper back that was photosensitive. The patient reported itching and burning of the affected skin. Past medical history included Raynaud’s phenomenon and CIDP for which he received prednisone and weekly infusions of IVIG. Infusions began approximately one year prior to the development of the rash, and symptoms flare after his weekly treatment. He noted improvement with the escalation of daily prednisone, but topical triamcinolone was ineffective.

**Figure 1:**
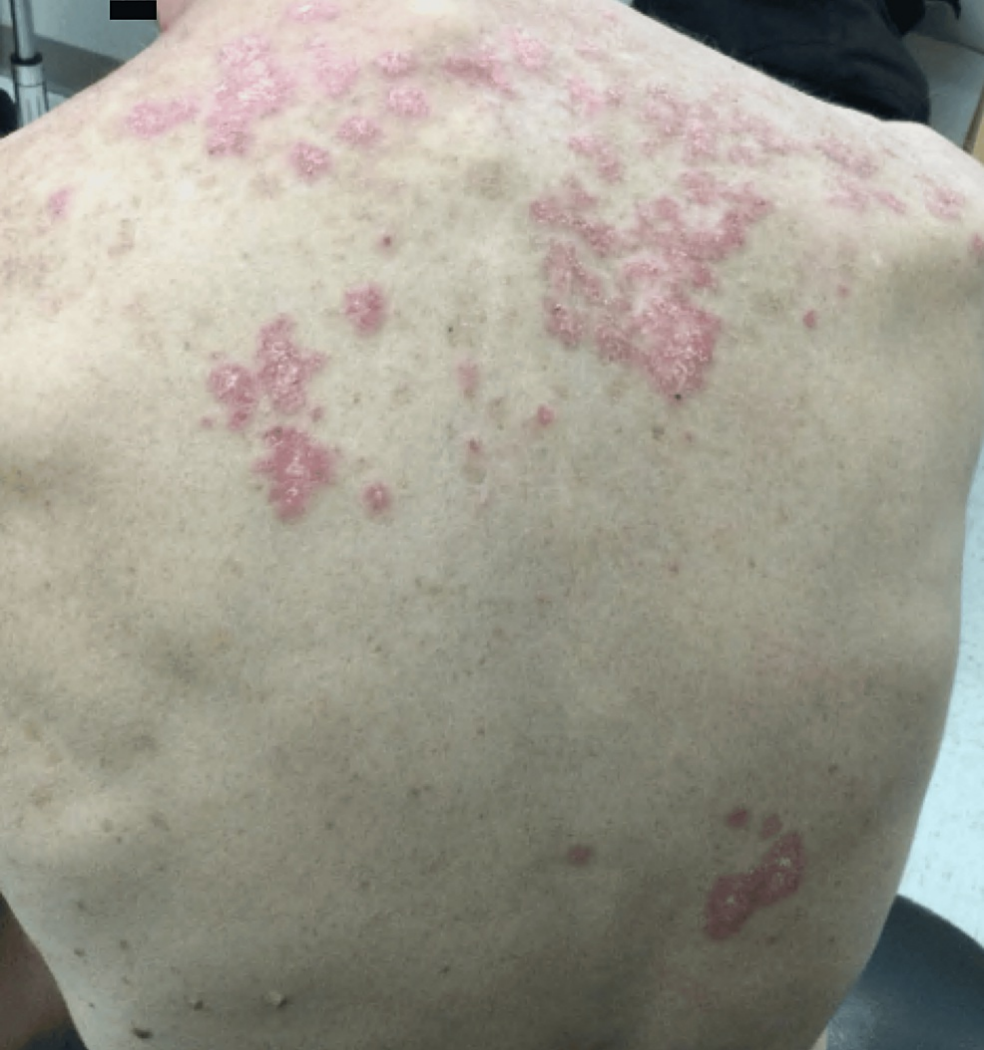
Erythematous papules that coalesce to form large plaques on the upper back.

**Figure 2:**
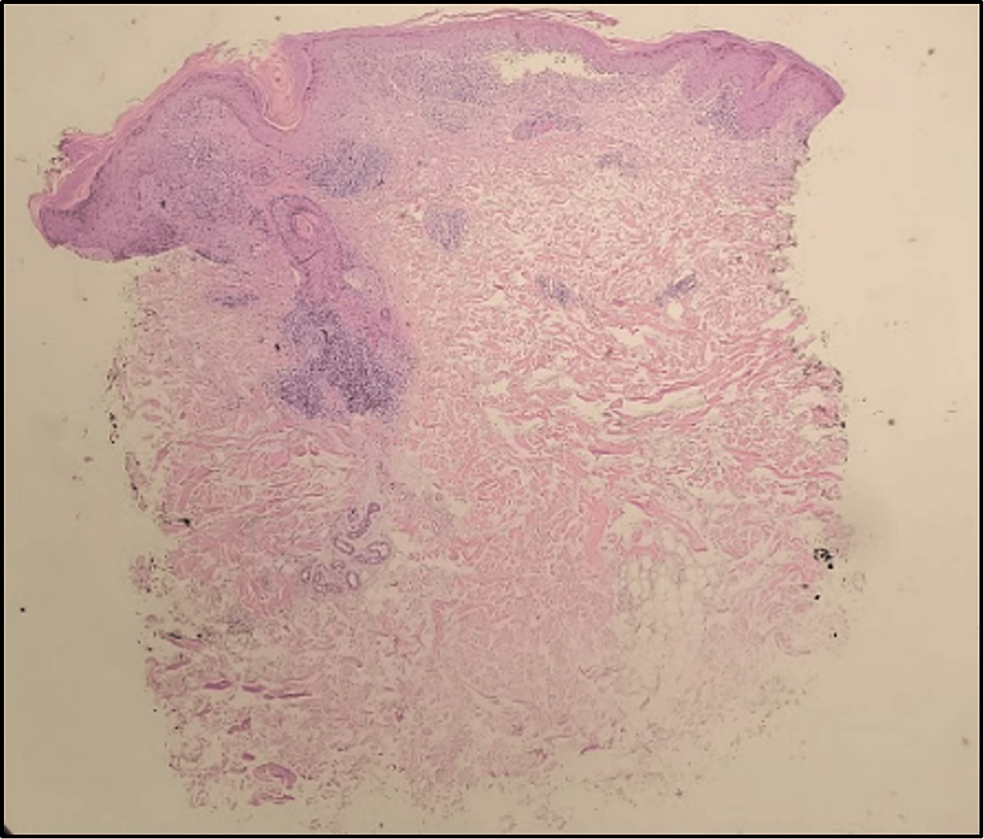
A punch biopsy at 40x magnification showing dense lichenoid infiltrate along the dermal-epidermal junction with perivascular and periadnexal accentuation and interstitial mucin deposition.

**Figure 3:**
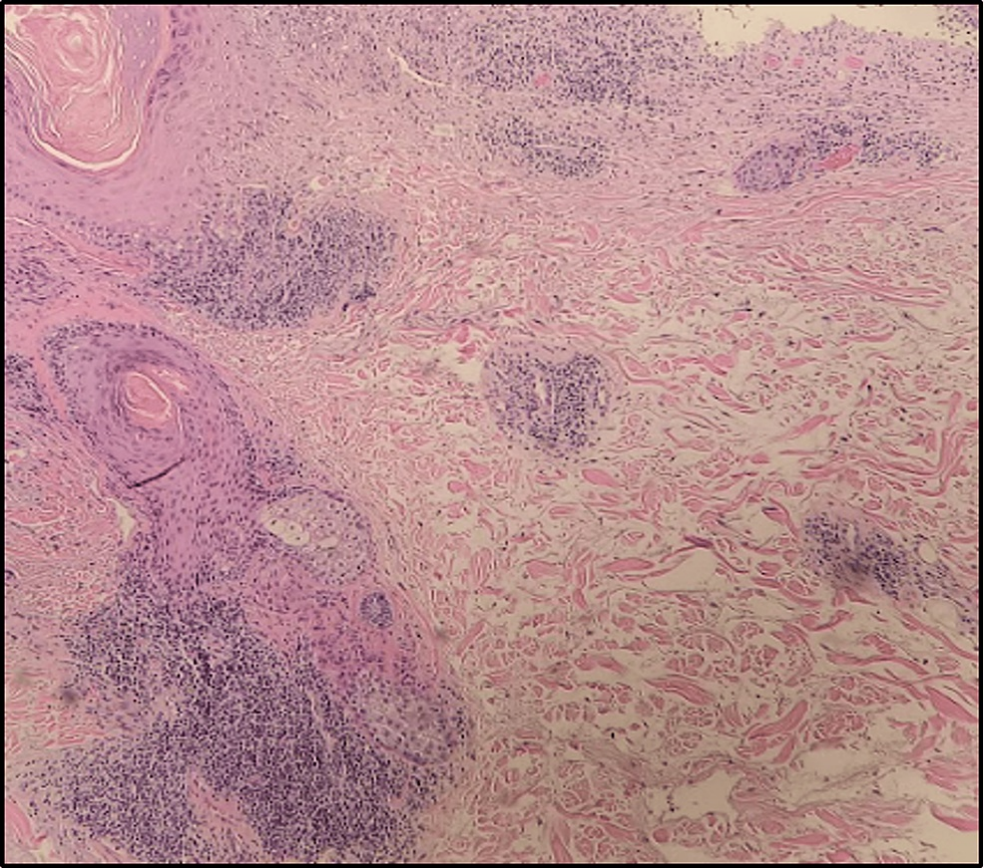
A punch biopsy at 100x magnification showing dense perivascular and periadnexal lymphocytic infiltrate and extensive interstitial mucin deposition.

Physical exam revealed erythematous, ovoid plaques on the shoulders, and an erythematous papulosquamous eruption which coalesced to form large patches on the chest, shoulders, and upper back (Figure 1). Scattered erythematous macules with fine scales were present on the scalp. A punch biopsy of the left trapezius revealed colloid bodies, a lichenified lymphocytic infiltrate along the dermal-epidermal junction, and interstitial deposits of mucin (Figures 2, 3).

Anti-neutrophilic antibodies (ANA) were weakly positive. Labs showed mild anemia and leukopenia with a hemoglobin of 12.4 g/dl and a white blood cell count of 3.22 k/cmm. Creatinine and GFR were within normal limits. Based on the clinical appearance, histopathological features, and patient’s history, a diagnosis of drug-induced SCLE was made. Following the diagnosis, neurology attempted to change the brand of IVIG from Gamunex (Grifols Therapeutics Inc., Barcelona, Spain) to Privigen (CSL Behring AG, Bern, Switzerland) but was unsuccessful due to medication shortages. The patient elected to continue management with Gamunex and prednisone since CIDP was controlled. Management is ongoing and hydroxychloroquine is being considered for his SCLE.

## Discussion

The pathophysiology of SCLE is multifactorial and not fully understood, but proposed mechanisms suggest ultraviolet light induces DNA damage and keratinocytes apoptosis, which leads to auto-antigen release and activation of innate and cell-mediated immune response [6]. Previous studies have suggested that drug-induced SCLE (DI-SCLE) may have a similar mechanism to idiopathic SCLE (I-SCLE), with the drug inducing DNA damage and auto-antigen release [12]. DI-SCLE is an important diagnosis to recall when presented with a patient with SCLE, as studies have shown up to a third of cases are drug-induced [13]. One investigation found that DI-SCLE was more likely to have widespread involvement with increased occurrence of bullous, vasculitic, or erythema multiforme-like lesions [14]. Another concluded that histopathological differences existed, with I-SCLE more likely to have mucin deposition and immunofluorescence findings, while DI-SCLE showed more leukocytic vasculitis [12]. While differentiating the two may be difficult, a lack of CLE symptoms before drug administration and resolution after drug cessation are indicative of DI-SCLE [1].

Diagnosis of SCLE depends on a combination of clinical, histopathological, and laboratory findings. As evidenced by cases like ours, a detailed medication history and a high index of suspicion are important because DI-SCLE can result from many medications and have a delayed presentation. The initial workup should include a thorough skin exam, lesion biopsy, and baseline ANA serology [1]. A routine complete blood count (CBC) and basic metabolic panel (BMP) should also be obtained to evaluate for systemic disease, which may be evidenced by abnormalities in kidney function or blood counts [1].

As discussed previously, there is a significant overlap between SLE and SCLE symptoms, so it should be on the differential for every SCLE patient. Chronic CLE should also be considered, particularly discoid lupus erythematosus (DLE). Like SCLE, DLE presents with annular, scaly plaques with an erythematous base and has similar histopathology [15]. It may be differentiated from SCLE based on anatomical involvement, with DLE often involving the face, ears, and scalp [6]. DLE is also likely to be followed by scarring and atrophy, which is absent in SCLE [1]. Furthermore, the clinical picture may be conflated, as 20% of SCLE patients may also have DLE [16].

There are currently no FDA-approved treatments for CLE, but commonly used regimens include topical corticosteroids, topical calcineurin inhibitors, antimalarials, and systemic steroids [17]. In most cases of DI-SCLE, the condition improves after withdrawal of the causative agent and full resolution is seen within weeks [8]. The prognosis is good, but patients may continue to have positive anti-Ro/SSA antibodies and may develop hypopigmented lesions or Sjogren’s disease [8,18]. The management of DI-SCLE may be complicated by preexisting conditions that necessitate treatment with the causative drug. Our patient had suboptimal control of his CIDP, which made it difficult to withdraw IVIG treatment entirely. This obstacle, along with the previously mentioned literature, guided the decision to change the brand of IVIG as a method of treatment.

A review of the literature reveals two previous publications in which CLE arose during IVIG treatment. In one case, a patient receiving IVIG for common variable immunodeficiency developed CLE with positive anti-Ro/SSA antibodies after five years of treatment [17]. This prompted an investigation of the IVIG preparations, which had high concentrations of anti-Ro/SSA antibodies, leading to the conclusion that passive transfer of antibodies played a role [19]. More recently, a case series of six patients with IVIG-induced SCLE in the setting of CIDP strongly suggests an association between IVIG and SCLE [10]. Three patients showed improvement after switching the brand of IVIG, and four saw improvement or remission with complete withdrawal of IVIG. This article proposed that photosensitivity induced by IVIG, which occurs in only ≤1% of patients, could trigger auto-antigen production and the development of SCLE [10].

## Conclusions

DI-SCLE can be caused by IVIG and commonly prescribed drugs, which makes it important for prescribers to consider prior to initiation for chronic diseases. If DI-SCLE develops, prompt recognition and rule-out of other causes can expedite the patient’s course. When possible, stopping the drug or switching the brand can lead to a full recovery. However, as in this case, stopping the causative drug is not always possible. In cases like these, interdepartmental management with dermatology and the prescribing physician is crucial.
